# Filtered Multitone Modulation Underwater Acoustic Communications Using Low-Complexity Channel-Estimation-Based MMSE Turbo Equalization

**DOI:** 10.3390/s19122714

**Published:** 2019-06-17

**Authors:** Lin Sun, Mei Wang, Guoheng Zhang, Haisen Li, Lan Huang

**Affiliations:** 1 College of Underwater Acoustic Engineering, Harbin Engineering University, Harbin 150001, China; hl1365034774@163.com; 2 School of Physics and Physical Engineering, Qufu Normal University, Qufu 273165, China; 3 Electro-Mechanical Engineering Institute, Shanghai 201109, China; meimeifeeler@sina.com; 4 Beijing Great Wall Electronic Equipment Co., Ltd, Beijing 100082, China; sea64094448@163.com; 5 Acoustic Science and Technology Laboratory, Harbin Engineering University, Harbin 150001, China; 6 Key Laboratory of Marine Information Acquisition and Security, Ministry of Industry and Information Technology, Harbin Engineering University, Harbin 150001, China

**Keywords:** underwater acoustic communications, filtered multitone modulation, interference suppression, turbo equalization, low-complexity channel-estimate-based minimum mean square error algorithm

## Abstract

Filtered multitone (FMT) modulation divides the communication band into several subbands to shorten the span of symbols affected by multipath in underwater acoustic (UWA) communications. However, there is still intersymbol interference (ISI) in each subband of FMT modulation degrading communication performance. Therefore, ISI suppression techniques must be applied to FMT modulation UWA communications. The suppression performance of traditional adaptive equalization methods often exploited in FMT modulation UWA communications is limited when the effect of ISI spans tens of symbols or large constellation sizes are used. Turbo equalization consisting of adaptive equalization and channel decoding can improve equalization performance through information exchanging and iterative processes. To overcome the shortcoming of traditional minimum mean square error (MMSE) equalization and effectively suppress the ISI with relatively low computation complexity, an FMT modulation UWA communication using low-complexity channel-estimation-based (CE-based) MMSE turbo equalization is proposed in this paper. In the proposed method, turbo equalization is first exploited to suppress the ISI in FMT modulation UWA communications, and the equalizer coefficients of turbo equalization are adjusted using the low-complexity CE-based MMSE algorithm. The proposed method is analyzed in theory and verified by simulation analysis and real data collected in the experiment carried out in a pool with multipath propagation. The results demonstrate that the proposed method can achieve better communication performance with a higher bit rate than the FMT modulation UWA communication using traditional MMSE adaptive equalization.

## 1. Introduction

The rapid rise of scientific and commercial activities in underwater environments has led to an increasing demand for high-rate and wide-band underwater acoustic (UWA) communications to sustain a mass of data transmission in UWA sensor networks. Multipath spread of UWA channels often is on the order of ten milliseconds [1,2,3], and therefore the high-rate and wide-band UWA communications using single-carrier (SC) modulation often have to adopt adaptive equalization with high computation complexity to suppress the intersymbol interference (ISI) spanning several tens or hundreds of symbols [3,4,5,6]. To reduce the span of ISI, multicarrier (MC) modulation has been used in UWA communications [7,8,9,10,11,12,13,14,15,16,17,18]. Based on whether the divided subbands are overlapping or not, the MC modulation used in UWA communications falls into two types. Orthogonal frequency division multiplexing (OFDM) is representative of the type with overlapping subbands [6,7,8,9,10,11,12]. In OFDM, the communication band is divided into hundreds of overlapping subbands with very narrow bandwidth that makes communication performance sensitive to frequency offset. Since the Doppler spread of UWA channels can lead to massive intercarrier interference (ICI), complicated techniques must be exploited in OFDM modulation UWA communications to suppress ICI. On the contrary, the MC modulation with non-overlapping subbands can avoid the problem of frequency offset sensitivity. According to inserting guard bands or not, the MC modulation with non-overlapping subbands can be further classified into two types. Compared to the type with guard bands, the type without guard bands can keep good balance between ICI suppression performance and spectral efficiency. Therefore, as the representation of MC modulation without overlapping subbands and guard bands, filtered multitone (FMT) modulation has attracted research interest and been applied to UWA communications in recent years [13,14,15,16,17]. In FMT modulation UWA communications, the span of ISI is obviously shortened through band splitting, but there is always some ISI due to the multipath spread of subchannels. To suppress the ISI, traditional minimum mean square error (MMSE) adaptive equalization is usually used in FMT modulation UWA communications [13,17]. 

For adaptive equalization, when the transmitted symbols are perfectly known in advance, the ISI can be completely eliminated. In practice, the transmitted symbols cannot be known beforehand, and therefore the performance of adaptive equalization is limited. Moreover, the UWA channel is a noisy channel, and the performance of adaptive equalization can also be degraded by noise. Turbo equalization is a technique that processes the received signal using adaptive equalization and channel decoding in an iterative fashion. In turbo equalization, adaptive equalization can exploit the last extrinsic information of channel decoding as a priori information to improve ISI suppression performance; channel decoding also can use the last extrinsic information of adaptive equalization as a priori information to further reduce noise. Since the ISI and noise are jointly suppressed, turbo equalization can overcome the shortcomings of adaptive equalization and has been used in UWA communications in recent decades [19,20,21,22,23,24,25,26]. Ideally, when adaptive equalization and channel decoding are both based on the maximum-likelihood (ML) or maximum a priori probability (MAP) algorithm, turbo equalization can achieve the best performance. However, the complexity of the ML/MAP algorithm can increase exponentially with the span of ISI, and therefore high computation complexity prohibits the application of ML/MAP-based turbo equalization to UWA communications. In practical UWA communications, turbo equalization based on the minimum mean square error (MMSE) algorithm is generally used in consideration of communication performance and computation complexity. MMSE turbo equalization can be classified into two types based on whether the estimated channel response is used in computing equalizer coefficients or not. Compared with the MMSE turbo equalization without channel estimation (CE), the CE-based MMSE turbo equalization requires a shorter training sequence and has faster convergence, but a slightly higher computation complexity due to the large-dimension matrix inversion in computing equalizer coefficients. To further reduce the computation complexity, the low-complexity CE-based MMSE turbo equalization is proposed and analyzed based on the terrestrial wireless channel model in [19]. Additionally, simulation analysis shows that this performance is nearly as good as that of the traditional CE-based MMSE turbo equalization [19]. 

At present, in FMT modulation UWA communications, the traditional MMSE adaptive equalization is a conventional approach used to suppress the ISI [13,17]. However, when the effect of ISI spans tens of symbols or large constellation sizes are used, the performance of FMT modulation UWA communications using traditional MMSE adaptive equalization is unacceptable. 

Considering the aforementioned advantages of turbo equalization and disadvantages of traditional MMSE adaptive equalization, the FMT modulation UWA communication using low-complexity CE-based MMSE turbo equalization is proposed in this paper. The proposed method applies turbo equalization to FMT modulation UWA communications to improve the ISI suppression performance. In the proposed method, error control coding (ECC) is used to encode the information bit subsequence before FMT modulation, and the low-complexity CE-based MMSE turbo equalization is adopted at the receiver to suppress ISI in each subsequence after FMT demodulation. The proposed method obtains good performance with relatively low computation complexity because the equalizer in turbo equalization is based on the low-complexity CE-based MMSE algorithm. The proposed method is analyzed in theory and verified by simulation results and data collected from a real experiment. The proposed method is compared with the FMT modulation UWA communications using traditional MMSE adaptive equalization, and the results show the validity of the proposed method.

The contribution of this paper is threefold: (1) turbo equalization is first applied to FMT modulation UWA communications to overcome the shortcomings of adaptive equalization; (2) the low-complexity CE-based MMSE algorithm is exploited to adjust the equalizer coefficients of turbo equalization to suppress ISI with relatively low computation complexity; (3) simulation and real experiments are designed to verify the proposed method.

This paper is organized as follows: in Section 2, the transmitter structure of the proposed method is briefly described. In Section 3, the receiver structure of the proposed method is presented and the principle of the low-complexity CE-based MMSE turbo equalization is analyzed in detail. In Section 4, the simulation and experiment results of the proposed method are presented, and the proposed method is compared with the results of the FMT modulation UWA communication using traditional MMSE adaptive equalization. Finally, the conclusion and summary is in Section 5.

## 2. Transmitter Structure

**Figure 1:**
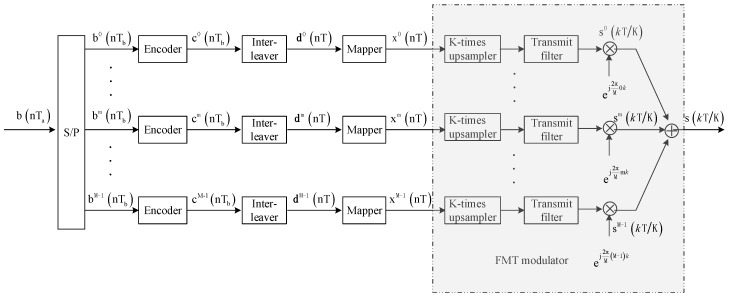
Block diagram for transmitter structure.

The transmitter structure of the proposed method is depicted based on the complex baseband model in Figure 1, where the number of transmit transducers and receiver hydrophones are both assumed to be one. It is shown that the information bit sequence $\left\{b\left(nT_{a}\right)\right\}$ with the time interval $T_{a}$ is firstly converted to several bit subsequences $\left\{b^{m}\left(nT_{b}\right)\right\},m=0,\cdots ,M-1$ by the serial-to-parallel (SP) converter, where $b^{m}\left(nT_{b}\right)\in \left\{0,1\right\}$ and $T_{b}=T_{a}/M$. Then, each bit subsequence is independently encoded by the encoder through inserting redundant bits into information bits to protect information bits from single bit errors or short burst errors. The encoders at the transmitter can be different or the same. Next, the coded bit subsequences $\left\{c^{m}\left(nT_{b}\right)\right\},m=0,\cdots ,M-1$ are interleaved, divided into Q-bit blocks $\left\{d^{m}\left(nT\right)\right\},m=0,\cdots ,M-1,T=Q\cdot T_{b}$ and mapped to the symbol sequences $\left\{x^{m}\left(nT\right)\right\},m=0,\cdots ,M-1$ from the 2^Q^-ary symbol alphabet $S=\left\{\alpha_{1},\alpha_{2},\cdots ,\alpha_{2^{Q}}\right\}$ with zero mean and unit energy, where $\alpha_{i}\in S$ is a complex number. Finally, the mapped symbol sequences are modulated by the FMT modulator consisting of *K*-times up-samplers, transmit filters, carrier modulators, and a combiner, and then the transmitted signal can be obtained. 

Referring to Figure 1, the transmitted signal can be expressed as:

(1) $$s\left(k\frac{T}{K}\right)=\sum_{m=0}^{M-1}\sum_{n=-\infty }^{+\infty }x^{m}\left(nT\right)g_{t}\left(k\frac{T}{K}-nT\right)e^{j\frac{2\pi }{M}mk}$$

where $x^{m}\left(nT\right)$ denotes the *n*-th symbol transmitted on the *m*-th subband, and $g_{t}\left(kT/K\right)$ denotes the time domain response of the transmit filter. In the FMT modulator, all transmit filters are the same and the selection of that must keep a balance between implementation complexity and spectral containment. In the proposed method, the root raised cosine (RRC) shaping filter is used as the transmit filter, and the value of the roll-off factor sets to α=K/M−1.

## 3. Receiver Structure 

### 3.1. FMT Demodulation

**Figure 2:**
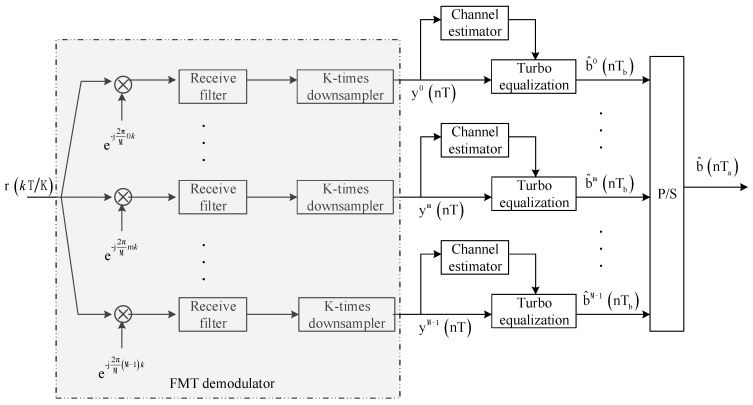
Block diagram for receiver structure.

The receiver structure of the proposed method is shown in Figure 2. After being transmitted through the UWA channel, the received signal, the input of the receiver, can be expressed as

(2) $$r\left(k\frac{T}{K}\right)=\sum_{p=0}^{N_{c}-1}s\left[\left(k-p\right)\frac{T}{K}\right]c\left(p\frac{T}{K}\right)+\eta \left(k\frac{T}{K}\right)$$

where $c\left(k\frac{T}{K}\right)$ denotes the discrete time domain response of the UWA channel with length $N_{c}$, and $\eta \left(k\frac{T}{K}\right)$ denotes the discrete sample of channel noise.

Combing Equation (1) with Equation (2), the received signal can be further expressed as

(3) $$r\left(k\frac{T}{K}\right)=\sum_{p=0}^{N_{c}-1}\sum_{m=0}^{M-1}\sum_{n=-\infty }^{+\infty }g_{t}\left[\left(k-p\right)\frac{T}{K}-nT\right]e^{j\frac{2\pi }{M}m\left(k-p\right)}c\left(p\frac{T}{K}\right)x^{m}\left(nT\right)+\eta \left(k\frac{T}{K}\right)$$

Referring to the receiver structure shown in Figure 2, the received signal is first processed by the FMT demodulator that consists of carrier demodulators, receiver filters, and *K*-times down-samplers. The *m*-th output signal of the FMT demodulator can be expressed as

(4) $$\begin{array}{ll} y^{m}\left(nT\right) & =\sum_{i=0}^{M-1}\sum_{l=-\infty }^{+\infty }\sum_{k=-\infty }^{+\infty }\sum_{p=0}^{N_{c}-1}g_{t}\left[\left(k-p\right)\frac{T}{K}\right]c\left(p\frac{T}{K}\right)e^{-j\frac{2\pi }{M}ip}g_{r}\left(nT-lT-k\frac{T}{K}\right)e^{j\frac{2\pi }{M}\left(i-m\right)\left(k+lK\right)}x^{i}\left(lT\right)\\ & +\sum_{k=-\infty }^{+\infty }\eta \left(k\frac{T}{K}\right)g_{r}\left(nT-k\frac{T}{K}\right)e^{-j\frac{2\pi }{M}mk} \end{array}$$

where $g_{r}\left(t\right)$ denotes the discrete time domain response of the receiver filter, that is matched to the transmit filter. 

Equation (4) can be further expressed as

(5) $$\begin{array}{ll} y^{m}\left(nT\right) & =\sum_{i=0}^{M-1}\sum_{l=-\infty }^{+\infty }h^{\left(m,i\right)}\left(nT-lT\right)x^{i}\left(lT\right)+w^{m}\left(nT\right)\\ & =h^{\left(m,m\right)}\left(0\right)x^{m}\left(nT\right)+\sum_{\begin{array}{l} l=-\infty \\ l\neq n \end{array}}^{+\infty }h^{\left(m,m\right)}\left(nT-lT\right)x^{m}\left(lT\right)\\ & +\sum_{\begin{array}{l} i=0\\ i\neq m \end{array}}^{M-1}\sum_{\begin{array}{l} l=-\infty \end{array}}^{+\infty }h^{\left(m,i\right)}\left(nT-lT\right)e^{j\frac{2\pi }{M}\left(i-m\right)lK}x^{i}\left(lT\right)+w^{m}\left(nT\right) \end{array}$$

where $h^{\left(m,i\right)}\left(nT\right)$ denotes the discrete time domain response of the subchannel between the *i*-th transmitted sequence $x^{i}\left(nT\right)$ and the *m*-th demodulated sequence $y^{m}\left(nT\right)$, and $w^{m}\left(nT\right)$ denotes the *i*-th output noise of the FMT demodulator. $h^{\left(m,i\right)}\left(nT\right)$ and $w^{m}\left(nT\right)$ can be expressed as

(6) $$\begin{array}{l} h^{\left(m,i\right)}\left(nT\right)=\sum_{i=0}^{M-1}\sum_{k=-\infty }^{+\infty }\sum_{p=0}^{N_{c}-1}g_{t}\left[\left(k-p\right)\frac{T}{K}\right]c\left(p\frac{T}{K}\right)e^{-j\frac{2\pi }{M}ip}g_{r}\left(nT-k\frac{T}{K}\right)e^{j\frac{2\pi }{M}\left(i-m\right)k}\\ w^{m}\left(nT\right)=\sum_{k=-\infty }^{+\infty }\eta \left(k\frac{T}{K}\right)g_{r}\left(nT-k\frac{T}{K}\right)e^{-j\frac{2\pi }{M}mk} \end{array}$$

It can be observed from Equation (5) that the interference in the demodulated signal $y^{m}\left(nT\right)$ includes three types, namely, the ISI from other symbols on the *m*-th subband, the ICI from symbol sequences on the other subbands, and the noise. In FMT modulation, the subbands are separated and have high spectral containment, and therefore the ICI is a minor concern even when the Doppler spread, caused by the water motion, exists in UWA communications [13,14,15,16,17]. This paper focus on exploiting turbo equalization to suppress the ISI in FMT modulation UWA communications. The ICI is slight, and therefore the effect of ICI is not taken into consideration. When the ICI is neglected, the demodulated signal $y^{m}\left(nT\right)$ can be expressed as:

(7) $$y^{m}\left(nT\right)=h^{\left(m,m\right)}\left(0\right)x^{m}\left(nT\right)+\sum_{\begin{array}{l} l=-\infty \\ l\neq n \end{array}}^{+\infty }h^{\left(m,m\right)}\left(nT-lT\right)x^{m}\left(lT\right)+w^{m}\left(nT\right)$$

After the output subsequences of turbo equalization are converted into a serial sequence, the estimated information bit sequence $\left\{\hat{b}\left(nT_{a}\right)\right\}$ can be obtained. The process of turbo equalization for each demodulated subsequence is described in Section 3.2.

### 3.2. Turbo Equalization 

#### 3.2.1. Procedure of Turbo Equalization 

**Figure 3:**
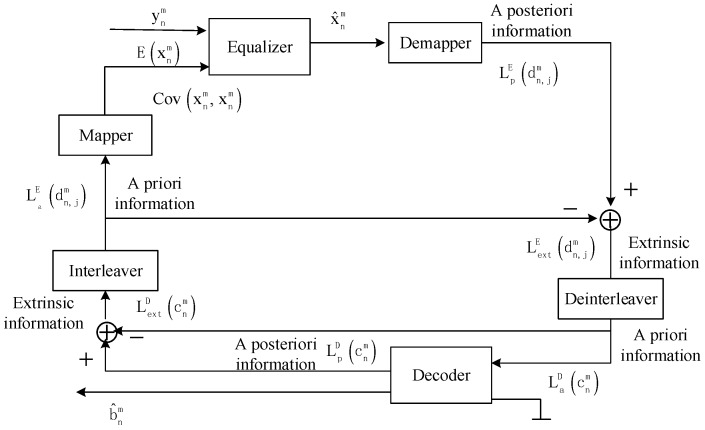
Block diagram for turbo equalization.

The procedure of turbo equalization is shown in Figure 3, where the soft information exchanged between the equalizer and the decoder is evaluated by the log likelihood ratio (LLR). For convenient expression, in Section 3.2, the symbol interval *T* and the bit interval *T_b_* are omitted, the discrete-time variate *n* is expressed as subscript, and the number of subsequence *m* is expressed as superscript. 

Referring to Figure 3, the demodulated subsequence $y_{n}^{m}$ is firstly sent to the equalizer with a priori information to estimate the mapped symbol ${\hat{x}}_{n}^{m}$. Then, the estimated symbol ${\hat{x}}_{n}^{m}$ is sent to the demapper to obtain the a posteriori LLR of bits belonging to the Q-bit block $L_{p}^{E}\left(d_{n,j}^{m}\right)$. In turbo equalization using the MMSE algorithm [19,22,24], the a posteriori LLR $L_{p}^{E}\left(d_{n,j}^{m}\right)$ can be expressed as

(8) $$\begin{array}{ll} L_{p}^{E}\left(d_{n,j}^{m}\right) & =\ln\frac{P\left(d_{n,j}^{m}=0|{\hat{x}}_{n}^{m}\right)}{P\left(d_{n,j}^{m}=1|{\hat{x}}_{n}^{m}\right)}\\ & =\ln\frac{\sum_{\forall d_{n}^{m}d_{n,j}^{m}=0}p\left({\hat{x}}_{n}^{m}|d_{n}^{m}\right)P\left(d_{n}^{m}\right)}{\sum_{\forall d_{n}^{m}:d_{n,j}^{m}=1}p\left({\hat{x}}_{n}^{m}|d_{n}^{m}\right)P\left(d_{n}^{m}\right)}\\ & =\underset{L_{ext}^{E}\left(d_{n,j}^{m}\right)}{\underset{︸}{\ln\frac{\sum_{\forall d_{n}^{m}:d_{n,j}^{m}=0}p\left({\hat{x}}_{n}^{m}|d_{n}^{m}\right)\prod_{\forall j^{\prime },j^{\prime }\neq j}P\left(d_{n,j^{\prime }}^{m}\right)}{\sum_{\forall d_{n}^{m}:d_{n,j}^{m}=1}p\left({\hat{x}}_{n}^{m}|d_{n}^{m}\right)\prod_{\forall j^{\prime },j^{\prime }\neq j}P\left(d_{n,j^{\prime }}^{m}\right)}}}+\underset{L_{a}^{E}\left(d_{n,j}^{m}\right)}{\underset{︸}{\ln\frac{P\left(d_{n,j}^{m}=0\right)}{P\left(d_{n,j}^{m}=1\right)}}} \end{array}$$

where $d_{n}^{m}=\left\{d_{n,1}^{m},d_{n,2}^{m},\cdots ,d_{n,Q}^{m}\right\}$ is the Q-bit block mapped to $x_{n}^{m}$, and $d_{n,j}^{m}$ is the *j*-th bit belonging to $d_{n}^{m}$. 

It is shown from Equation (8) that both the a priori LLR $L_{a}^{E}\left(d_{n,j}^{m}\right)$ and the extrinsic information $L_{ext}^{E}\left(d_{n,j}^{m}\right)$ are included in the a posteriori LLR $L_{p}^{E}\left(d_{n,j}^{m}\right)$. In turbo equalization, to avoid fast convergence, only the extrinsic information is exchanged between the equalizer and the decoder. Equalization and decoding are repeated until the performance convergence is obtained. For the initial process of turbo equalization, neither the equalizer nor the decoder has any a priori information.

#### 3.2.2. Low-Complexity CE-Based MMSE Algorithm 

In this section, the low-complexity CE-based MMSE algorithm used in the proposed method is discussed in detail [19]. In turbo equalization, the equalizer can be a linear equalizer (LE) or a decision feedback equalizer (DFE). The DFE using hard decision suffers from error propagation. The DFE using soft decision can avoid error propagation, but computation complexity is relatively high. Hence, in consideration of performance and computation complexity, the LE is used in our proposed method. 

To facilitate understanding, some frequently used notations are first introduced. Vectors and matrices are denoted by bold lowercase and bold uppercase letters, respectively. The i×j matrix $0_{i\times j}$ contains all zeros, and $I_{i}$ is the i×i identity matrix. The operator diag(⋅) denotes transferring a length i vector into a i×i square matrix with the vector elements along the diagonal. The operator ${\left(\cdot \right)}^{T}$ denotes conjugate transpose. The operator E(⋅) denotes expectation. The operator $\mathrm{cov}\left(x,y\right)=E\left(xy^{H}\right)-E\left(x\right)E\left(y^{H}\right)$ denotes covariance, where ${\left(\cdot \right)}^{H}$ denotes complex conjugate transpose. 

Based on the exact CE-based MMSE algorithm, the estimation ${\hat{x}}_{n}^{m}$ of the LE can be expressed as

(9) $${\hat{x}}_{n}^{m}=E\left(x_{n}^{m}\right)+\mathrm{cov}\left(x_{n}^{m},y_{n}^{m}\right)\mathrm{cov}{\left(y_{n}^{m},y_{n}^{m}\right)}^{-1}\left(y_{n}^{m}-E\left(y_{n}^{m}\right)\right)$$

where $y_{n}^{m}=\left[y_{n-N_{2}}^{m},y_{n-N_{2}+1}^{m},\cdots y_{n+N_{1}}^{m}\right]$ denotes the input observation vector of the equalizer, $N_{1}$ and $N_{2}$ denote the number of noncausal and causal equalizer coefficients, and the total equalizer coefficient is $N=N_{1}+N_{2}+1$. 

Assuming the discrete time composited channel response $h^{\left(m,m\right)}\left(n\right)$ shown in Equation (6) has L taps, the observation $y_{n}^{m}$ can be expressed as

(10) $$y_{n}^{m}=H^{m}x_{n}^{m}+{\left[w_{n-N_{2}}^{m},w_{n-N_{2}+1}^{m},\cdots ,w_{n+N_{1}}^{m}\right]}^{T}$$

where $x_{n}^{m}={\left[x_{n-N_{2}-L+1}^{m},x_{n-N_{2}-L+2}^{m},\cdots ,x_{n+N_{1}}^{m}\right]}^{T}$, and $H^{m}$ is the N×(N+L−1) channel convolution matrix

(11) $$H^{m}=\left[\begin{array}{cccccccc} h_{L-1}^{m} & h_{L-2}^{m} & \cdots & h_{0}^{m} & 0 & \cdots & & 0\\ 0 & h_{L-1}^{m} & h_{L-2}^{m} & \cdots & h_{0}^{m} & 0 & \cdots & 0\\ & & & \ddots & & & & \\ 0 & & \cdots & 0 & h_{L-1}^{m} & h_{L-2}^{m} & \cdots & h_{0}^{m} \end{array}\right]$$

In the proposed method, the transmitted symbols are assumed to be equiprobable and independent identically distributed (IID). In order to let the estimation ${\hat{x}}_{n}^{m}$ be independent of the a priori LLR $d_{n,j}^{m}\in d_{n}^{m}$, the a priori LLR of each bit belonging to $d_{n}^{m}$ must be set to 0 when ${\hat{x}}_{n}^{m}$ is computed, which results in $E\left(x_{n}^{m}\right)=0$ and $C\mathrm{ov}\left(x_{n}^{m},x_{n}^{m}\right)=1$. Assuming the channel noise samples $\eta \left(\frac{k}{K}\right)$ are IID Gaussian noise samples with a mean of zero, the estimation ${\hat{x}}_{n}^{m}$ can be further expressed as

(12) $${\hat{x}}_{n}^{m}={\left(f_{n}^{m}\right)}^{H}\left(y_{n}^{m}-E\left(y_{n}^{m}\right)+s_{}^{m}{\bar{x}}_{n}^{m}\right)$$

where

(13) $$\begin{array}{l} E\left(y_{n}^{m}\right)=H^{m}E\left(x_{n}^{m}\right)=H^{m}{\left[{\bar{x}}_{n-L-N_{2}+1}^{m},{\bar{x}}_{n-L-N_{2}+2}^{m},\cdots ,{\bar{x}}_{n+N_{1}}^{m}\right]}^{T}\\ f_{n}^{m}=C\mathrm{ov}{\left(y_{n}^{m},y_{n}^{m}\right)}^{-1}s_{}^{m}\\ \mathrm{Cov}\left(y_{n}^{m},y_{n}^{m}\right)=\delta_{\eta }^{2}I_{N}+H^{m}\mathrm{Cov}\left(x_{n}^{m},x_{n}^{m}\right){\left(H^{m}\right)}^{H}+\left(1-v_{n}^{m}\right)s_{}^{m}{\left(s_{}^{m}\right)}^{H}\\ s_{}^{m}=H^{m}{\left[0_{1\times \left(N_{2}+L-1\right)}\;1\;0_{1\times \left(N_{1}\right)}\right]}^{T}\\ \mathrm{Cov}\left(x_{n}^{m},x_{n}^{m}\right)=diag\left(v_{n-L-N_{2}+1}^{m},v_{n-L-N_{2}+2}^{m},\cdots ,v_{n+N_{1}}^{m}\right) \end{array}$$

where $\delta_{\eta }^{2}$ denotes the variance of noise samples $\eta \left(\frac{k}{K}\right)$, ${\bar{x}}_{n}^{m}$ and $v_{n}^{m}$ denotes the mean and variance of $x_{n}^{m}$. The values of ${\bar{x}}_{n}^{m}$ and $v_{n}^{m}$ can be computed using the a priori LLR $L_{a}^{e}\left(d_{n,j}^{m}\right)$

(14) $$\begin{array}{l} {\bar{x}}_{n}^{m}=\sum_{\alpha_{i}\in S}\alpha_{i}\cdot P\left(x_{n}^{m}=\alpha_{i}\right)=\sum_{\alpha_{i}\in S}\alpha_{i}\cdot \prod_{j=1}^{Q}P\left(d_{n,j}^{m}=d\right),d\in \left\{0,1\right\}\\ v_{n}^{m}=E\left[{\left(x_{n}^{m}\right)}^{2}\right]-{\left[E\left(x_{n}^{m}\right)\right]}^{2}=\bar{{\left(x_{n}^{m}\right)}^{2}}-{\left|{\bar{x}}_{n}^{m}\right|}^{2}\\ P\left(d_{n,j}^{m}=d\right)=\frac{1}{2}\left(1+\left(2d-1\right)\tanh\left[\frac{L_{a}^{e}\left(d_{n,j}^{m}\right)}{2}\right]\right) \end{array}$$

where $P\left(d_{n,j}^{m}=d\right)$ denotes the probability of $d_{n,j}^{m}=d$, and $P\left(x_{n}^{m}=\alpha_{i}\right)$ denotes the probability of $x_{n}^{m}=\alpha_{i}$.

From Equation (12) to Equation (14), it can be observed that in linear turbo equalization using the exact CE-based MMSE algorithm, $f_{n}^{m}$ must be updated with the computation of ${\hat{x}}_{n}^{m}$ due to the a priori LLR $L_{a}^{e}\left(d_{n,j}^{m}\right)$ varying with n. The direct computation complexity of $f_{n}^{m}$ increases with $O\left(N^{3}\right)$, even when the fast recursive solution is used, the computation complexity of $f_{n}^{m}$ still has $O\left(N^{2}\right)$. That is to say, when the exact CE-based MMSE algorithm is used, the computation complexity of each symbol in equalization of each iterative process is $O\left(N^{2}\right)$. To achieve good performance with lower computation complexity, a low-complexity CE-based MMSE algorithm proposed in [19] is adopted in the proposed method. 

Based on the low-complexity CE-based MMSE algorithm, the filter coefficients $f_{n}^{m}$ are computed using the average of $\mathrm{Cov}\left(y_{n}^{m},y_{n}^{m}\right)$

(15) $$\begin{array}{l} {\bar{f}}_{}^{m}={\left(\delta_{\eta }^{2}I_{N}+H^{m}{\bar{V}}^{m}{\left(H^{m}\right)}^{H}+\left(1-{\bar{v}}_{}^{m}\right)s_{}^{m}{\left(s_{}^{m}\right)}^{H}\right)}^{-1}s_{}^{m}\\ {\bar{V}}^{m}=\frac{1}{N_{x}}\sum_{n=1}^{N_{x}}\mathrm{Cov}\left(x_{n}^{m},x_{n}^{m}\right)\\ {\bar{v}}_{}^{m}=\frac{1}{N_{x}}\sum_{n=1}^{N_{x}}v_{n}^{m} \end{array}$$

where $N_{x}$ denotes the total number of the transmitted symbols. 

Then, the estimation ${\hat{x}}_{n}^{m}$ can be computed by

(16) $${\hat{x}}_{n}^{m}={\left({\bar{f}}_{}^{m}\right)}^{H}\left(y_{n}^{m}-E\left(y_{n}^{m}\right)+s_{}^{m}{\bar{x}}_{n}^{m}\right)$$

Defining a new vector $f_{}^{m}$

(17) $$f_{}^{m}={\left(\delta_{\eta }^{2}I_{N}+H^{m}{\bar{V}}^{m}{\left(H^{m}\right)}^{H}\right)}^{-1}s_{}^{m}$$

The vector ${\bar{f}}_{}^{m}$ can be expressed by $f_{}^{m}$ as follows

(18) $$\begin{array}{l} {\bar{f}}_{}^{m}=K\cdot f_{}^{m}\\ K={\left(1+\left(1-{\bar{v}}^{m}\right){\left(s^{m}\right)}^{H}f_{}^{m}\right)}^{-1} \end{array}$$

Based on Equations (16)–(18), the mean and variance of ${\hat{x}}_{n}^{m}$ can be computed by

(19) $$\begin{array}{cc} \mu_{n,i}^{m} & =K\cdot {\left(f_{}^{m}\right)}^{H}\left(E\left(y_{n}^{m}|x_{n}^{m}=\alpha_{i}\right)-E\left(y_{n}^{m}\right)+s_{}^{m}{\bar{x}}_{n}^{m}\right)=K\cdot \alpha_{i}{\left(f_{}^{m}\right)}^{H}s_{}^{m}\\ {\left(\sigma_{n,i}^{m}\right)}^{2} & =K^{2}\cdot {\left(f^{m}\right)}^{H}C\mathrm{ov}\left(y_{n}^{m},y_{n}^{m}|x_{n}^{m}=\alpha_{i}\right)f_{}^{m}\\ & =K^{2}\cdot {\left(f_{}^{m}\right)}^{H}\left(\delta_{\eta }^{2}I_{N}+H^{m}{\bar{V}}^{m}{\left(H^{m}\right)}^{H}-{\bar{v}}_{}^{m}s_{}^{m}{\left(s_{}^{m}\right)}^{H}\right)f_{}^{m} \end{array}$$

Combing Equation (19) with Equation (8), the extrinsic information $L_{ext}^{E}\left(d_{n,j}^{m}\right)$ is given by

(20) $$\begin{array}{ll} L_{ext}^{E}\left(d_{n,j}^{m}\right) & =\ln\frac{\sum_{\forall s_{i}:s_{n,j}=0}p\left({\hat{x}}_{n}^{m}|d_{n}^{m}=s_{i}\right)\prod_{\forall j^{\prime },j^{\prime }\neq j}P\left(d_{n,j^{\prime }}^{m}=s_{i,j^{\prime }}^{}\right)}{\sum_{\forall s_{i}:s_{n,j}=1}p\left({\hat{x}}_{n}^{m}|d_{n}^{m}=s_{i}\right)\prod_{\forall j^{\prime },j^{\prime }\neq j}P\left(d_{n,j^{\prime }}^{m}=s_{i,j^{\prime }}^{}\right)}\\ & =\ln\frac{\sum_{\forall s_{i}:s_{n,j}=0}\exp\left(-\rho_{n,i}^{m}+\prod_{\forall j^{\prime },j^{\prime }\neq j}s_{i,j^{\prime }}^{}L\left(d_{n,j}^{m}\right)/2\right)}{\sum_{\forall s_{i}:s_{n,j}=1}\exp\left(-\rho_{n,i}^{m}+\prod_{\forall j^{\prime },j^{\prime }\neq j}s_{i,j^{\prime }}^{}L\left(d_{n,j}^{m}\right)/2\right)} \end{array}$$

where $s_{i}=\left\{s_{i,1},s_{i,2}\cdots ,s_{i,Q}\right\}$ is the bit pattern corresponding to the symbol $\alpha_{i}$ belonging to the symbol alphabet $S=\left\{\alpha_{1},\alpha_{2},\cdots ,\alpha_{2^{Q}}\right\}$, and $\rho_{n,i}^{m}$ can be computed using

(21) $$\rho_{n,i}^{m}=\frac{{\left|{\hat{x}}_{n}^{m}-\mu_{n,i}^{m}\right|}^{2}}{{\left(\sigma_{n,i}^{m}\right)}^{2}}=\frac{{\left|{\left(f_{}^{m}\right)}^{H}\left(y_{n}^{m}-E\left(y_{n}^{m}\right)+s_{}^{m}{\bar{x}}_{n}^{m}\right)-\alpha_{i}{\left(f_{}^{m}\right)}^{H}s_{}^{m}\right|}^{2}}{{\left(f_{}^{m}\right)}^{H}\left(\delta_{\eta }^{2}I_{N}+H^{m}{\bar{V}}^{m}{\left(H^{m}\right)}^{H}-{\bar{v}}_{}^{m}s_{}^{m}{\left(s_{}^{m}\right)}^{H}\right)f_{}^{m}}$$

The term ${\bar{V}}^{m}$ in Equation (21) can be further approximated by ${\bar{v}}_{}^{m}I_{N+L-1}$ and that does not degrade the performance substantially when the number of symbols $N_{x}$ is large. Substituting ${\bar{V}}_{}^{m}$ with ${\bar{v}}_{}^{m}I_{N+L-1}$, the $f_{}^{m}$ shown in Equation (17) is simplified to

(22) $${\hat{f}}_{}^{m}={\left(\delta_{\eta }^{2}I_{N}+{\bar{v}}_{}^{m}H^{m}{\left(H^{m}\right)}^{H}\right)}^{-1}s_{}^{m}$$

Substituting ${\hat{f}}_{}^{m}$ for $f_{}^{m}$ in Equation (21), the new expression of $\rho_{n,i}^{m}$ can be expressed as

(23) $$\begin{array}{ll} \rho_{n,i}^{m} & =\frac{{\left|{\left({\hat{f}}_{}^{m}\right)}^{H}\left(y_{n}^{m}-E\left(y_{n}^{m}\right)+s_{}^{m}{\bar{x}}_{n}^{m}\right)-\alpha_{i}{\left({\hat{f}}_{}^{m}\right)}^{H}s_{}^{m}\right|}^{2}}{{\left({\hat{f}}_{}^{m}\right)}^{H}\left(\delta_{\eta }^{2}I_{N}+{\bar{v}}_{}^{m}H^{m}{\left(H^{m}\right)}^{H}-{\bar{v}}_{}^{m}s_{}^{m}{\left(s_{}^{m}\right)}^{H}\right){\hat{f}}_{}^{m}}\\ & =\frac{{\left|{\left({\hat{f}}_{}^{m}\right)}^{H}\left(y_{n}^{m}-E\left(y_{n}^{m}\right)+s_{}^{m}{\bar{x}}_{n}^{m}\right)-\alpha_{i}{\left({\hat{f}}_{}^{m}\right)}^{H}s_{}^{m}\right|}^{2}}{{\left({\hat{f}}_{}^{m}\right)}^{H}s_{}^{m}\left(1-{\left(s_{}^{m}\right)}^{H}{\hat{f}}_{}^{m}\right)} \end{array}$$

Combining Equation (23) with Equation (20), the extrinsic information of the equalizer using the low-complexity CE-based MMSE algorithm can be finally computed. 

It can be observed from Equations (20)–(23) that the equalizer coefficients ${\hat{f}}_{}^{m}$ keep constant in equalization of each iterative process. Therefore, in turbo equalization using the low-complexity CE-based MMSE algorithm, the computation complexity of each symbol in equalization of each iterative process is reduced to O(N), and that is much smaller than the computation complexity $O\left(N^{2}\right)$ in turbo equalization using the exact MMSE algorithm.

## 4. Performance Assessment 

### 4.1. Simulation Analysis

#### 4.1.1. Simulation Setup

**Figure 4:**
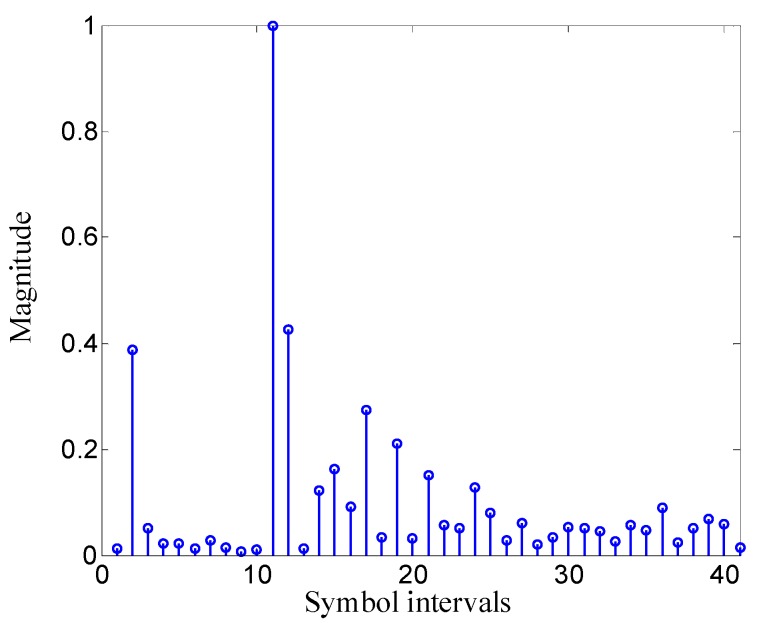
Discrete-time channel response used in simulation analysis.

The UWA channel is one of the most complicated channels, and there is still no closed formula to express it. To verify the validity of the proposed method in UWA environments, a real UWA channel response measured in the experiment carried out in September 2014 at Songhua Lake, Jilin, China, is used for simulation analysis. In the Songhua Lake experiment, the transmit sensor and the receiver hydrophone were suspended from two ships moored on the shore. The transmit sensor was deployed 1 m below the surface, and the receiver hydrophone was placed 0.5 m below the surface. The water depth at the transmitter and the receiver was about 4 m, and the communication distance was 350 m. The measured discrete-time channel response is shown in Figure 4. The FMT modulation UWA communication using traditional MMSE adaptive equalization was also analyzed in the same simulation condition for comparison with the proposed method. To express conveniently, the FMT modulation UWA communication using traditional MMSE adaptive equalization is abbreviated to the method using traditional MMSE adaptive equalization. The parameters for simulation analysis are shown in Table 1.

#### 4.1.2. Simulation Results

In simulation analysis, bit error rate (BER), symbol error rate (SER), and output mutual information (MI) are used as performance indicators. The mutual information (MI) can be computed using

(24) $$MI\left(L_{o};X\right)=\frac{1}{2}\sum_{x\in \left\{-1,1\right\}}\int_{-\infty }^{+\infty }f_{L_{o}}\left(l|x\right)\cdot \log_{2}\frac{2f_{L_{o}}\left(l|x\right)}{f_{L_{o}}\left(l|+1\right)+f_{L_{o}}\left(l|-1\right)}dl$$

where $L_{o}$ denotes the output LLRs of the equalizer or the decoder in the proposed method, and $f_{L_{o}}\left(l|x\right)$ denotes the conditional probability distribution function that can be estimated through observing the histogram of the output LLRs. The value of MI ranges from 0 to 1. MI = 0 denotes no a priori information, and MI = 1 denotes perfect a priori information. MI not only can reflect the reliability of the output LLR, but also can be used to determine the iteration number of turbo equalization. When the output LLR of the decoder is close to 1, the performance of turbo equalization reaches convergence, and the iterative process can be terminated.

**Figure 5:**
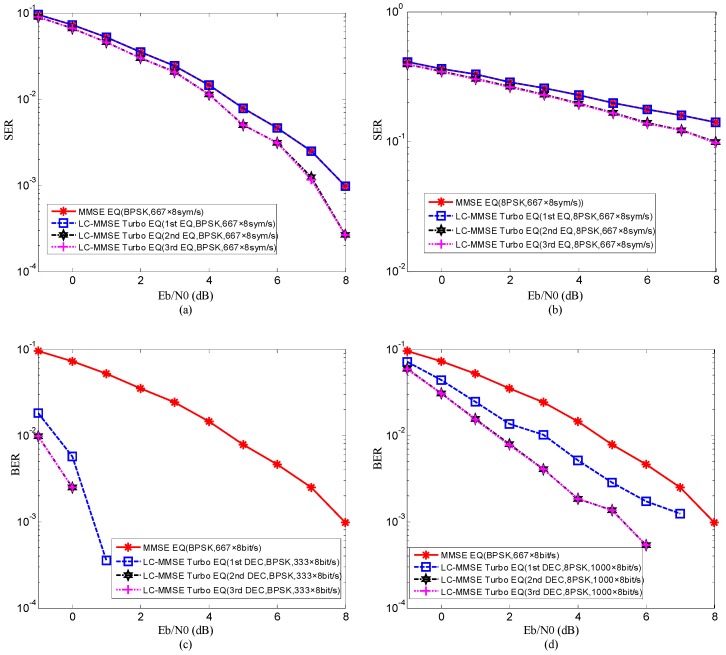
Simulation results of the proposed method and the method using traditional MMSE adaptive equalization: (**a**) and (**b**) the symbol error rate (SER) of low-complexity channel estimation (CE)-based MMSE equalization in the proposed method and the method using traditional MMSE adaptive equalization; (**c**) and (**d**) the bit error rate (BER) after decoding in the proposed method and the method using traditional MMSE adaptive equalization.

**Figure 6:**
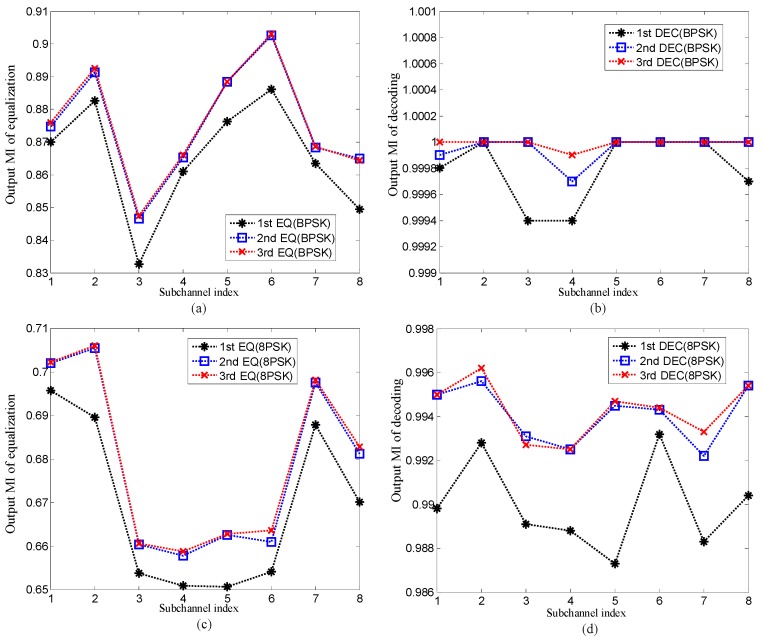
The output mutual information (MI) of the proposed method for $E_{b}/N_{0}=2\mathrm{dB}$: (**a**) and (**c**) the output MI of equalization; (**b**) and (**d**) the output MI of decoding.

The SER and BER of the proposed method and the output MI after equalization and decoding in the proposed method for $E_{b}/N_{0}=2\mathrm{dB}$ are shown in Figure 5 and Figure 6. The performance of the method using traditional MMSE adaptive equalization is also shown in Figure 5 for comparison with the proposed method. In Figure 5, the signal to noise ratio (SNR) can be computed by $E_{b}/N_{0}=\sum_{k=0}^{L-1}{\left|h_{k}^{m}\right|}^{2}/2\delta_{\eta }^{2}Q$. 

Several observations can be obtained through Figure 5 and Figure 6. Firstly, when the same $E_{b}/N_{0}$ is used, the performance of the method using traditional MMSE adaptive equalization is equal to that of the first equalization of the proposed method. The reason for this observation is that in the first equalization of the proposed method, the average ${\bar{v}}_{}^{m}=\frac{1}{N_{x}}\sum_{n=1}^{N_{x}}v_{n}^{m}$ equals 1 due to the a priori LLR of the equalizer $L_{a}^{e}\left(d_{n,j}^{m}\right)=0$, and therefore the coefficient vector ${\hat{f}}_{}^{m}$ shown in Equation (22) changes to ${\hat{f}}_{}^{m}={\left(\delta_{\eta }^{2}I_{N}+H^{m}{\left(H^{m}\right)}^{H}\right)}^{-1}s_{}^{m}$ which is the solution of the traditional MMSE adaptive equalization. It means that the traditional MMSE adaptive equalization is only the first step of the low-complexity CE-based MMSE turbo equalization. Secondly, in the proposed method, the performance of the second equalization and decoding is obviously superior to that of the first equalization and decoding. The reason is that in the second equalization and decoding, benefitting from the extrinsic information exchanging in turbo equalization, the a priori LLR of the equalizer and decoder are no longer zeros, and therefore the BER and SER are reduced. Thirdly, compared with the second equalization and decoding, the performance improvement after the third equalization and decoding is not obvious. The reason is that after the second equalization and decoding, the output MI of the decoder is very close to 1 as shown in Figure 6, and therefore the additional iterative process cannot improve the performance since the communication performance has reached convergence. Fourthly, when mapping patterns are the same, compared with the method using traditional MMSE adaptive equalization, the proposed method achieves better performance after convergence with bit rate reduction by half. The reason for this observation is that the 1/2 rate convolutional code used in turbo equalization can improve the communication performance, but it also leads to the reduction of transmission efficiency. Lastly, when different mapping patterns are adopted, the proposed method with 8 phase shift keying (8PSK) mapping achieves better communication performance at the higher bit rate than the method using traditional MMSE adaptive equalization with BPSK mapping. The reason for this observation is that the iteration process of turbo equalization and the extrinsic information exchanging in the proposed method and can further suppress interference and improve communication performance.

The above simulation results illustrate that the performance of the proposed method is superior to that of the method using traditional MMSE adaptive equalization.

### 4.2. Experiment

#### 4.2.1. Experimental Setup

**Figure 7:**
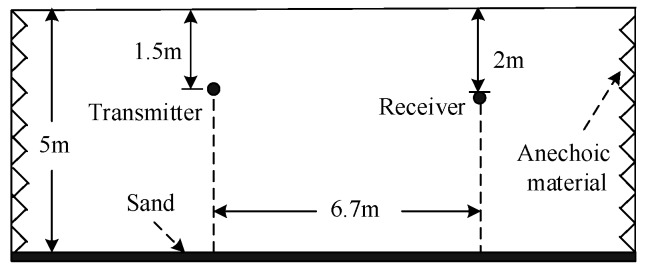
Experiment configuration.

To further assess the validity of the proposed method, the experiment was designed and carried out in an indoor pool with four sides covered with acoustic anechoic materials. The length, width, and depth of the pool were 45 m, 6 m, and 5 m, respectively. The bottom of the pool was covered with sand, and both the surface and bottom of the pool could reflect the acoustic signal. In the experiment, the transmit sensor deployed at 1.5 m below the surface was a hemispherical transducer, and the receive sensor placed at 2 m below the surface was a spherical hydrophone. The communication distance was 6.7 m. The experiment configuration is shown in Figure 7.

**Figure 8:**
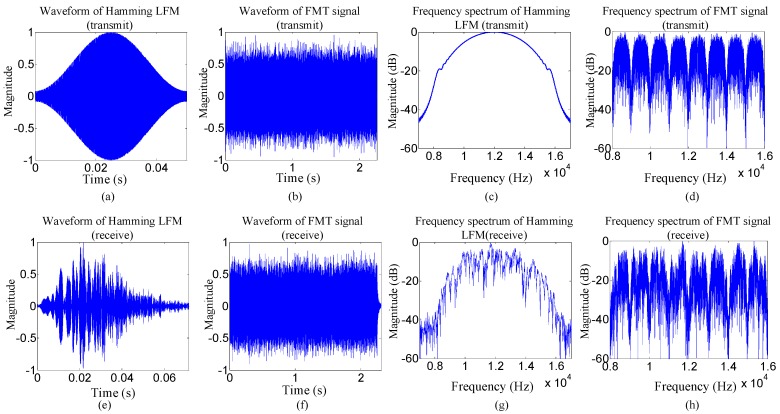
Time domain waveforms and frequency spectrums of linear frequency modulated (LFM) signals with a hamming window and FMT signals at the transmitter and the receiver on the condition of 8PSK mapping scheme used: (**a**–**d**) time domain waveforms and frequency spectrums of the LFM signal and the FMT signal at the transmitter; (**e**–**h**) time domain waveforms and frequency spectrums of the LFM signal and the FMT signal at the receiver.

The parameters used in the experiment are the same as those shown in Table 1. The transmit signal was organized in the packet structure consisting of the probe signal, the 100 ms guard time interval, and the FMT signal. The probe signal used for frame synchronization was a 50 ms and an 8–16 kHz linear frequency modulated (LFM) signal with a hamming window. The FMT signal includes 1500 symbols and ahead 100 symbols were used to train equalizer coefficients. When the 8PSK mapping scheme is used, time domain waveforms and frequency spectrums of the LFM signal with a hamming window and the FMT signal at the transmitter and the receiver, as shown in Figure 8. Time domain waveforms and frequency spectrums of the LFM signal and the FMT signal at the receiver are also shown in Figure 8 for comparison. The SNR at the receiver is 23 dB. In Figure 8, all time domain waveforms and frequency spectrums are normalized, and the received FMT signal is processed by frame synchronization.

Two observations can be obtained from Figure 8. Firstly, the LFM signal and the FMT signal are obviously distorted by the multipath effect after passing through the UWA channel, and therefore ISI suppression technique must be used to deal with the ISI in the received FMT signal for good communication performance. Secondly, although the distortion appears in the frequency spectrum of the received FMT signal, the adjacent subbands still keep unoverlapping, and therefore each output subsequence of the FMT demodulator can be individually processed using turbo equalization.

#### 4.2.2. Channel Response Estimation

**Figure 9:**
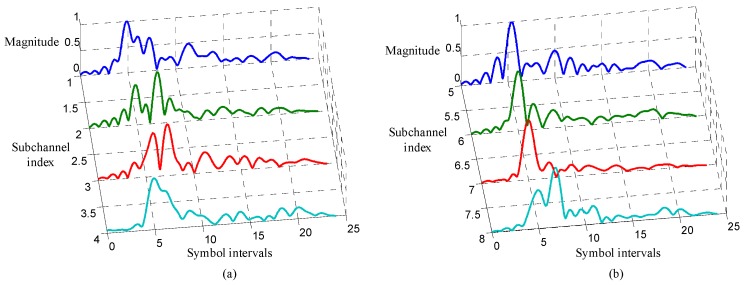
Subchannel responses: (**a**) responses of the first four subchannels; (**b**) responses of the last four subchannels.

In the turbo equalization using the low-complexity CE-based MMSE algorithm, each subchannel response in Equation (6) must be first estimated. In the experiment, the subchannel responses are estimated using the recursive least square (RLS) algorithm with the forgetting factor 0.999, and the filter for channel estimation has $N_{1}=5$ noncausal coefficients and $N_{2}=18$ causal coefficients. The estimated subchannel responses are shown in Figure 9. It can be observed from Figure 9 that the ISI span of each subchannel is about 20 symbol intervals.

#### 4.2.3. Experiment Results 

The experiment results of the proposed method are shown in Table 2 and Table 3. The experiment results of the method using traditional MMSE adaptive equalization are shown in Table 4. For clear comparison, the BERs of the proposed method with 8PSK mapping and the method using traditional adaptive equalization with BPSK mapping are listed in Table 5. Note that the symbol rate of the proposed method is the same as that of the reference method. The iteration of the turbo equalization terminates when the output MI of decoding is over 0.99.

Through comparing the results in Table 2, Table 3, Table 4 and Table 5, several observations can be obtained. Firstly, the SER of the method using traditional MMSE adaptive equalization is equal to that of the first equalization of the proposed method. Secondly, the proposed method realizes error-free transmission only through one time equalization and decoding for BPSK mapping, while two times equalization and decoding is needed to reach performance convergence for 8PSK mapping. Thirdly, when the same mapping pattern is used, although the proposed method achieves better performance after convergence, the information bit rate has been cut down to half of that in the method using traditional MMSE equalization. Lastly, as shown in Table 5, when a different mapping pattern is used, the proposed method with 8PSK mapping can achieve better communication performance at a higher bit rate than the method using traditional MMSE adaptive equalization with BPSK mapping. All experiment results are consistent with the simulation analysis. 

**Figure 10:**
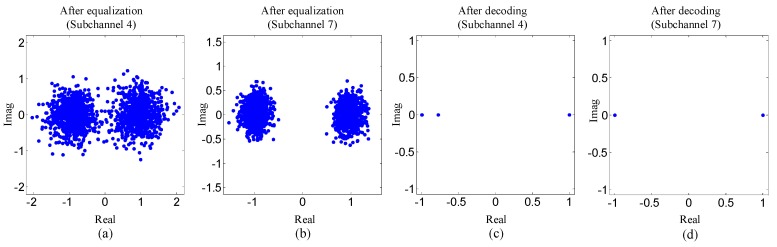
Scatterplots for subband four and seven of the proposed method with BPSK mapping: (**a**) and (**b**) the scatterplots indicating the estimated symbols after equalization; (**c**) and (**d**) the scatterplots indicating the mean of the symbols after decoding.

**Figure 11:**
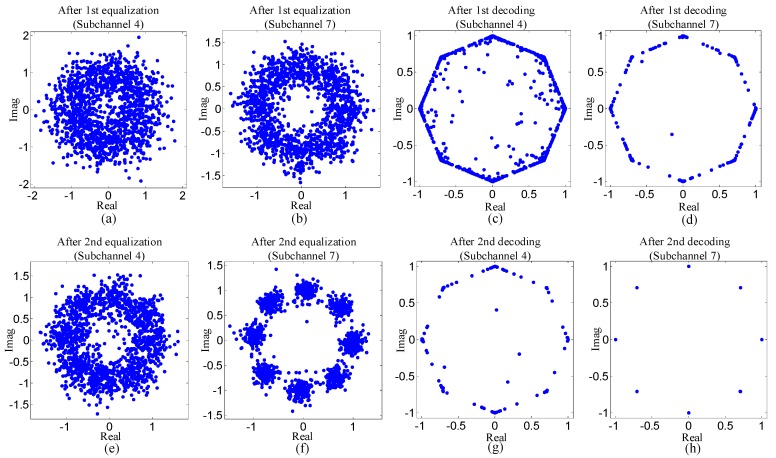
Scatterplots for subband four and subband seven of the proposed method with 8PSK mapping: (**a**), (**b**), (**e**) and (**f**) the scatterplots indicating the estimated symbols after equalization; (**c**), (**d**), (**g**) and (**h**) the scatterplots indicating the mean of the symbols after decoding.

To reflect experiment results visually, Figure 10 and Figure 11 show the scatterplots of the proposed method. Two types of scatterplots are shown in Figure 10 and Figure 11. One type indicates the estimated symbols after equalization, and the other reflects the mean of symbols that is computed using the a posteriori LLR of the decoder. 

Three observations can be obtained from Figure 10 and Figure 11. Firstly, the dots in the scatterplots corresponding to subchannel seven are much closer to the desired values than that of subchannel four. Secondly, for each subchannel, the dots in the scatterplots become more centralized around the desired values after decoding. Thirdly, in Figure 11, the dots in the scatterplots after the second equalization are much nearer the desired values than that in the scatterplots after the first equalization. The three observations above are all consistent with the results shown in Table 2 and Table 3.

## 5. Conclusions

In FMT modulation UWA communications, although the span of ISI has been reduced through band splitting, there is still a shortened ISI which needs to be suppressed. The performance of traditional adaptive equalization commonly exploited in FMT modulation UWA communications is limited when the effect of ISI spans tens of symbols or large constellation sizes are used. In order to further suppress the ISI with relatively low computation complexity, the FMT modulation UWA communication using low-complexity CE-based MMSE turbo equalization is proposed in this paper. The proposed method is analyzed in theory and verified by simulation analysis based on a measured channel response, and a real trial carried out in a pool with multipath propagation. Benefitting from the error correction of ECC, the information exchanged between decoding and equalization, and the iterative process, the ISI can be effectively suppressed. The results show that the proposed method achieves better communication performance at a higher bit rate than the FMT modulation UWA communication using traditional MMSE adaptive equalization. 

## Figures and Tables

**Table 1:** Parameters for simulation analysis.

Parameters	The Proposed Method	The Method Using Traditional Minimum Mean Square Error (MMSE) Equalization
Communication band B (kHz)	8–16	8–16
The number of subbands of filtered multitone (FMT) modulation M	8	8
Roll-off factor of each transmit filter α	0.5	0.5
Symbol interval T (ms)	1.5	1.5
Mapping pattern	Binary phase shift keying (BPSK) 8 phase shift keying (8PSK)	BPSK, 8PSK
Number of information bits on each subband	750 (BPSK), 2250 (8PSK)	1500 (BPSK), 4500 (8PSK)
Error control coding (ECC)	Convolution code with generator polynomial [7,5]	None
Number of mapped symbols on each subband	1500	1500
Symbol rate on each subband (symbol/s)	667	667
Information bit rate on each subband (bit/s)	333 (BPSK), 1000 (8PSK)	667 (BPSK), 2000 (8PSK)
The number of equalization coefficients	25	25

**Table 2:** Results of the proposed method with BPSK mapping.

Band (kHz)	After Equalization (Each Subband: 667 symbol/s)		After Decoding (Each Subband: 333 bit/s)	
	SER	MI	BER	MI
8–9	0/1400	1	0/700	1
9–10	3/1400	0.997	0/700	1
10–11	1/1400	0.999	0/700	1
11–12	4/1400	0.989	0/700	1
12–13	0/1400	0.999	0/700	1
13–14	0/1400	1	0/700	1
14–15	0/1400	1	0/700	1
15–16	3/1400	0.998	0/700	1

**Table 3:** Results of the proposed method with 8PSK mapping.

Band (kHz)	After Equalization (Each Subband: 667 symbol/s)				After Decoding (Each Subband: 1000 bit/s)			
	SER		MI		BER		MI	
	1st	2nd	1st	2nd	1st	2nd	1st	2nd
8–9	411/1400	39/1400	0.739	0.971	18/2100	0/2100	0.931	0.998
9–10	144/1400	8/1400	0.908	0.991	0/2100	0/2100	0.990	0.999
10–11	240/1400	11/1400	0.830	0.991	0/2100	0/2100	0.973	0.999
11–12	496/1400	263/1400	0.700	0.939	55/2100	5/2100	0.887	0.991
12–13	390/1400	39/1400	0.734	0.961	29/2100	0/2100	0.920	0.996
13–14	206/1400	3/1400	0.849	0.994	4/2100	0/2100	0.978	0.999
14–15	205/1400	17/1400	0.889	0.994	6/2100	0/2100	0.983	1
15–16	338/1400	13/1400	0.765	0.951	9/2100	0/2100	0.954	0.995

**Table 4:** Results of the method using traditional MMSE adaptive equalization.

Band (kHz)	SER of BPSK Mapping (Each Subband: 667 symbol/s, 667 bit/s)	SER of 8PSK Mapping (Each Subband: 667 symbol/s, 2000 bit/s)
8–9	0/1400	411/1400
9–10	3/1400	144/1400
10–11	1/1400	240/1400
11–12	4/1400	496/1400
12–13	0/1400	390/1400
13–14	0/1400	206/1400
14–15	0/1400	205/1400
15–16	3/1400	338/1400

**Table 5:** Comparison between the proposed method and the method using traditional adaptive equalization in BER.

Band (kHz)	BER of the Proposed Method after Performance Convergence Reached (Each Subband: 667 symbol/s, 1000 bit/s)	BER of the Method Using Traditional Adaptive Equalization (Each Subband: 667 symbol/s, 667 bit/s)
8–9	0/2100	0/1400
9–10	0/2100	3/1400
10–11	0/2100	1/1400
11–12	5/2100	4/1400
12–13	0/2100	0/1400
13–14	0/2100	0/1400
14–15	0/2100	0/1400
15–16	0/2100	3/1400
Total	5/16800	11/11200

## References

[1] Brumm H., Slabbekoorn H. (2005). Acoustic communication in noise. Adv. Study Behav..

[2] Stojanovic M., Preisig J. (2009). Underwater acoustic communication channels: Propagation models and statistical characterization. IEEE Commun. Mag..

[3] Singer A.C., Nelson J.K., Kozat S.S. (2009). Signal processing for underwater acoustic communications. IEEE Commun. Mag..

[4] Stojanovic M., Catipovic J., Proakis J.G. (1993). Adaptive multichannel combining and equalization for underwater acoustic communications. J. Acoust. Soc. Am..

[5] Song H.C., Hodgkiss W.S. (2013). Efficient use of bandwidth for underwater acoustic communication. J. Acoust. Soc. Am..

[6] Sun D., Liu L., Cui H., Zhang Y. (2015). Single-carrier underwater acoustic communication combined with channel shortening and dichotomous coordinate descent recursive least squares with variable forgetting factor. IET Commun..

[7] Mason S., Berger C.R., Zhou S., Willett P. (2008). Detection, synchronization, and Doppler scale estimation with multicarrier waveforms in underwater acoustic communication. IEEE J. Sel. Areas Commun..

[8] Kang T., Iltis A.R. (2008). Iterative carrier frequency offset and channel estimation for underwater acoustic OFDM systems. IEEE J. Sel. Areas Commun..

[9] Radosevic A., Ahmed R., Duman T.M., Proakis J.G., Stojanovic M. (2014). Adaptive OFDM modulation for underwater acoustic communications: Design considerations and experimental results. IEEE J. Ocean. Eng..

[10] Skinder Z., Szczepanek M., Wilczewski E. (2015). Differentially coherent multichannel detection of acoustic OFDM signals. IEEE J. Ocean. Eng..

[11] Wan L., Zhou H., Xu X., Huang Y., Zhou S., Shi Z., Cui J.H. (2015). Adaptive modulation and coding for underwater acoustic OFDM. IEEE J. Ocean. Eng..

[12] Chi W., Yin J., Huang D., Zielinski A. (2015). Experimental demonstration of differential OFDM underwater acoustic communication with acoustic vector sensor. Appl. Acoust..

[13] Gomes J., Stojanovic M. Performance analysis of filtered multitone modulation systems for underwater communication. Proceedings of the IEEE International Conference on Oceans.

[14] Li H.S., Sun L., Du W.D., Zhou T., Chen B.W. (2017). Multiple-input multiple-output passive time reversal acoustic communication using filtered multitone modulation. Appl. Acoust..

[15] Amini P., Chen R.R., Farhang-boroujeny B. (2015). Filterbank multicarrier communications for underwater acoustic channels. IEEE J. Ocean. Eng..

[16] Silva L., Gomes J. Sparse Channel estimation and equalization for underwater filtered multitone. Proceedings of the IEEE International Conference on Oceans.

[17] Sun L., Chen B.W., Li H.S., Zhou T. (2015). Time reversal acoustic communication using filtered multitone modulation. Sensors.

[18] Zhang G.S., Dong H.F. (2011). Experimental assessment of a multicarrier underwater acoustic communication system. Appl. Acoust..

[19] Tuchler M., Singer A.C., Koetter R. (2002). Minimum mean squared error equalization using a priori information. IEEE Trans. Signal Process..

[20] Yang Z., Zheng Y.R. (2016). Iterative Channel estimation and turbo equalization for multiple-input multiple-output underwater acoustic communications. IEEE J. Ocean. Eng..

[21] Zheng Y.R., Wu J., Xiao C. (2015). Turbo equalization for single-carrier underwater acoustic communications. IEEE Commun. Mag..

[22] Tuchler M., Singer A.C. (2011). Turbo equalization: An overview. IEEE Trans. Inf. Theory.

[23] Choi J.W. (2011). Adaptive linear turbo equalization over doubly selective channels. IEEE J. Ocean. Eng..

[24] Tao J., Wu J., Zheng Y.R., Xiao C. (2011). Enhanced MIMO LMMSE turbo equalization: Algorithm, simulations, and undersea experimental results. IEEE Trans. Signal Process..

[25] Otnes R., Eggen T.H. (2008). Underwater acoustic communications: Long-term test of turbo equalization in shallow water. IEEE J. Ocean. Eng..

[26] Duan W., Zheng Y.R. (2016). Bidirectional soft-decision feedback turbo equalization for MIMO systems. IEEE Trans. Veh. Technol..

